# Safety and validity of extracorporeal fenestration and in situ fenestration in patients with aortic disease involving the left subclavian artery: a prospective, single-center, randomized controlled study

**DOI:** 10.1186/s13063-025-08746-5

**Published:** 2025-01-30

**Authors:** Xiaojian Jia, Jingjin Wu, Caiyou Ding, Yanbo Lou

**Affiliations:** Department of Vascular Surgery, The Fourth Affiliated Hospital, Zhejiang University School of Medicine, Yiwu, Zhejiang China

**Keywords:** Aortic disease, Extracorporeal fenestration, In situ fenestration, Thoracic endovascular aortic repair, Randomized controlled study, Study protocol

## Abstract

**Background:**

Thoracic aortic pathologies involving the aortic arch are a great challenge for vascular surgeons. Maintaining the patency of supra-aortic branches while excluding the aortic lesion remains difficult. Thoracic EndoVascular Aortic Repair (TEVAR) with fenestrations provides a feasible and effective approach for this type of disease. The main purpose of this trial is to assess the safety and validity of extracorporeal fenestration and in situ fenestration in patients with aortic disease involving the left subclavian artery.

**Methods:**

This is a prospective, single-center, randomized controlled study. A total of 170 eligible patients will be recruited from The Fourth Affiliated Hospital, Zhejiang University School of Medicine in China and randomized on a 1:1 basis either to the group A (extracorporeal fenestration) or the group B (in situ fenestration). The primary outcome will be the all-cause mortality (30 days). The secondary outcomes will include incidence of secondary intervention (30 days, 6 months, 1 year), incidence of endoleak (30 days, 6 months, 1 year), incidence of major adverse events (MAE) (i.e., immediate procedural success and complications) (30 days, 6 months, 1 year), immediate technical success rate, and all-cause mortality (6 months, 1 year).

**Discussion:**

Suppose extracorporeal fenestration non-inferior to in situ fenestration in patients with aortic disease involving the left subclavian artery. This trial aims to demonstrate the safety and validity of extracorporeal fenestration and in situ fenestration in patients with aortic disease involving the left subclavian artery, which is expected to provide a reference for Thoracic EndoVascular Aortic Repair (TEVAR) with fenestrations.

**Trial registration:**

ClinicalTrials.gov NCT06256757. Registered on February 5, 2024. https://clinicaltrials.gov/study/NCT06256757.

## Administrative information

Note: the numbers in curly brackets in this protocol refer to SPIRIT checklist item numbers. The order of the items has been modified to group similar items (see http://www.equator-network.org/reporting-guidelines/spirit-2013-statement-defining-standard-protocol-items-for-clinical-trials/).

**Table:** 

Title {1}	Safety and validity of extracorporeal fenestration and in situ fenestration in patients with aortic disease involving the left subclavian artery: a prospective, single-center, randomized controlled study
Trial registration {2a and 2b}	Registered at ClinicalTrials.gov: NCT06256757; date of registration: February 5, 2024, https://clinicaltrials.gov/study/NCT06256757
Protocol version {3}	Protocol version 1.0, dated January 7, 2024
Funding {4}	There are currently no funding sources in the list.
Author details {5a}	First author: Xiaojian Jia Corresponding author: Yanbo Lou The authors are all from the Department of Vascular Surgery, The Fourth Affiliated Hospital, Zhejiang University School of Medicine, Yiwu, China. XJ conceived and designed the study. JJ and CY contributed to study design and to development of the proposal. YB was the lead trial methodologist. All authors read and approved the final manuscript.
Name and contact information for the trial sponsor {5b}	Sponsor: The Fourth Affiliated Hospital, Zhejiang University School of Medicine Contact name: Mr. L Address: N1 Shangcheng Avenue, Yiwu, Zhejiang Province Email: yanbo_lou@zju.edu.cn
Role of sponsor {5c}	Yanbo Lou all contributed to the design and management of this study.

## Introduction

### Background and rationale {6a}

Thoracic aortic pathologies involving the aortic arch are a great challenge for vascular surgeons. Maintaining the patency of supra-aortic branches while excluding the aortic lesion remains difficult. Endovascular repair of the aortic arch using manufactured fenestrated and or branched devices seems to enjoy a satisfactory level of technical success in many cases together with a progressively reduced load in terms of early death [1]. Thoracic EndoVascular Aortic Repair (TEVAR) with fenestrations provides a feasible and effective approach for this type of disease and has gradually become the most used approach in these patients [2, 3]. In situ fenestration as feasible and with a high rate of technical success and satisfactory results in the short term [4–6]. Previous studies suggested that the viability of preserving LSA blood flow in TEVAR using extracorporeal fenestration to treat various aortic pathologies in various clinical situations and the technical feasibility and short-term results that may justify the use of this method in emergency cases [7]. A systematic review and meta-analysis showed favorable short-term results of both extracorporeal fenestration and in situ fenestration techniques with low mortality and strokes rates [8]. But a study demonstrated that the snare double loop reinforcement has an advantage regarding durability of the graft branch assembly. Moreover, non-reinforced fenestrations show signs of weakness and lack of stability, which questions the in situ fenestration procedures [9]. However, most of the previous studies were retrospective, extracorporeal fenestration or in situ fenestration techniques which is the best choice were unknown. The main purpose of this trial is to assess the safety and validity of extracorporeal fenestration and in situ fenestration in patients with aortic disease involving the left subclavian artery.

## Objectives {7}

The main purpose of this trial is to assess the safety and validity of extracorporeal fenestration and in situ fenestration in patients with aortic disease involving the left subclavian artery.

## Trial design {8}

The study design is a prospective, single-center, non-inferiority, randomized controlled trial. A total of 170 eligible participants will be divided randomly into two groups in a ratio of 1:1.

**Fig. 1:**
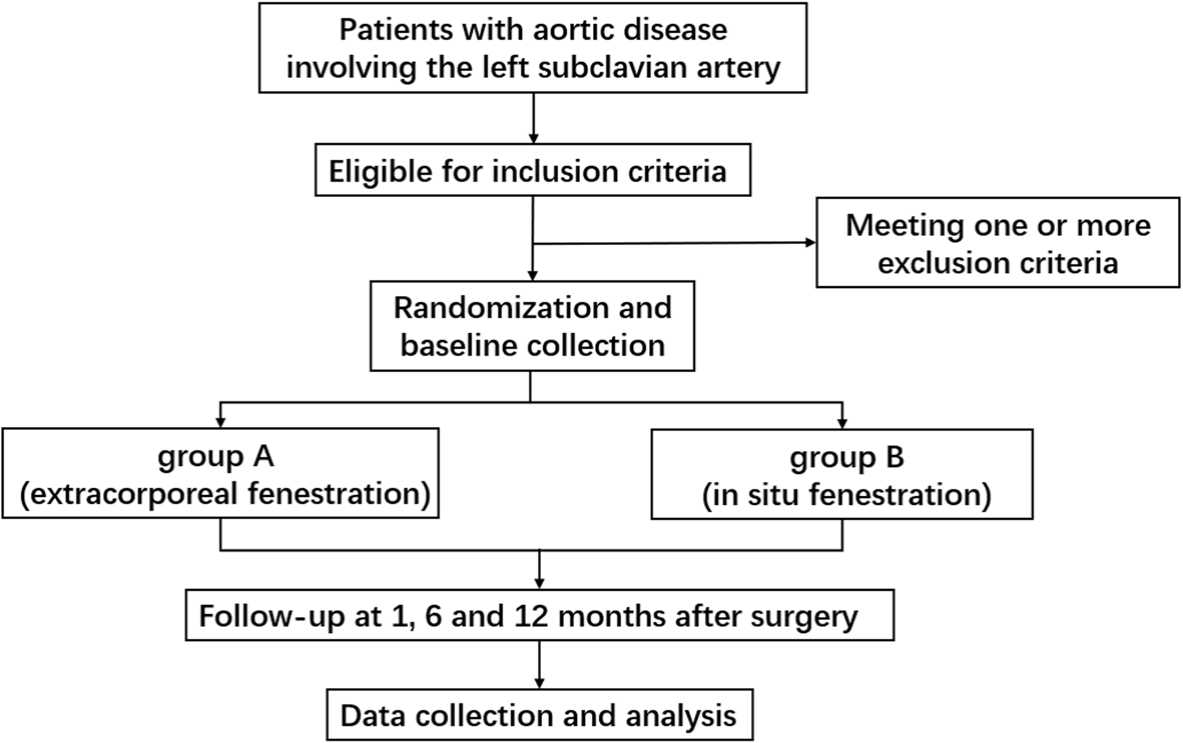
Flowchart of the trial

The flowchart of the trial is presented in Fig. 1.

## Methods: participants, interventions, and outcomes

### Study setting {9}

The current study will take place in The Fourth Affiliated Hospital of Zhejiang University School of Medicine in China.

### Eligibility criteria {10}

Inclusion criteria (all of the following criteria were met for enrolment):

1. Patients aged greater than 18 years old and less than 80 years old.
2. Patients diagnosed with thoracic aortic disease, including thoracic aortic dissection, thoracic aortic aneurysm, thoracic aortic ulcer, and intramural hematoma.
3. Patients needed endovascular repair (TEVER).
4. Patients who needed complete coverage of the left subclavian artery (planned landing zone was in zone 2 of the aortic zone).
5. The proximal lesion involved the proximal aorta within 1.5 cm of the posterior edge of the left subclavian artery (LSA) opening, but not the left common carotid artery (LCCA).
6. Suitable femoral artery, iliac artery, and brachial artery access can be used for endovascular treatment.
7. The patients could understand the purpose of the study, volunteered to participate in the study, and informed consent was signed by the subjects themselves or their legal representatives.
8. Patients were willing to undergo follow-up evaluation as required by the study protocol.
9. The life expectancy of the patient is more than 12 months.

Exclusion criteria (if any of the following criteria is met):

1. The lesion involved the proximal aorta of the LCCA opening (zone 0 and zone 1).
2. The patient has a definite connective tissue disease (e.g., Marfan syndrome).
3. The subjects had a history of previous TEVAR.
4. Patients who have had or may have had severe allergic reactions to contrast media (anaphylactic shock, exfoliative dermatitis, etc.).
5. Patients with contraindications to antiplatelet and anticoagulant drugs.
6. The patient’s compliance was poor and the follow-up could not be expected on time.
7. Patients with acute systemic infection.
8. Patients cannot tolerate general anesthesia.
9. Patients judged by the investigator to be ineligible for endovascular treatment.

### Who will take informed consent? {26a}

This trial will be conducted in patients with thoracic aortic disease diagnosed in The Fourth Affiliated Hospital of Zhejiang University School of Medicine from May 2024. All participants included in the trial signed an informed consent form.

### Additional consent provisions for collection and use of participant data and biological specimens {26b}

N/A. No collection or use of participant data or biospecimens was performed.

## Interventions

### Explanation for the choice of comparators {6b}

TEVAR is a feasible and effective approach for aortic disease, which will be implemented in all groups. TEVAR with fenestrations provides a feasible and effective approach for patients with aortic disease involving the left subclavian artery.

### Intervention description {11a}

Eligible patients will be randomized to either the group A (extracorporeal fenestration) or the group B (in situ fenestration). Participants will be done the scheduled endovascular repair which will be performed by surgeons with at least 5 years’ experience. The Chinese expert consensus serves as a technical reference [10].

#### In situfenestration

Left subclavian artery (LSA): From the left brachial artery (LBA), a 6F angle-adjustable sheath (Lifetech, Inc., Shenzhen, China) is introduced retrogradely until its tip reaches the aortic stent graft. The tip is then adjusted to be as perpendicular as possible to the larger curve of the aortic stent graft. Once the sheath gets to the ideal position, a flexible needle (21 gauge, Futhrough, Lifetech, Inc.) is employed to create the fenestration in the aortic stent graft (Ankura, Lifetech, Inc., Shenzhen, China). Following the puncture, a 0.018-inch guidewire (V-18 ControlWire; Boston Scientific, Natick, MA) is inserted through the needle aperture and into the ascending aorta [11–13]. After needle fenestration, the initial aperture is expanded using balloon angioplasty. Deployment of stent in the branch artery.

#### Extracorporeal fenestration

Based on the preoperative CTA reconstructions, the diameter of the aorta and branch vessels, lengths, angles to the arch, clock positions, and related relationships are measured, and a preoperative design for the fenestrations is developed. The outer sheath of the stent graft (Ankura, Lifetech, Inc., Shenzhen, China) is then pushed back for several centimeters under sterile conditions, allowing the proximal portion of the stent graft to be released [14]. The length of the released segment should be 1 to 2 cm distal from the location of fenestration [14]. Using a sterile ruler, the location of the fenestration is determined in accordance with the preoperative plan. The 12 o’clock position is considered to be at the front of the trigger. The position of the stent graft relative to the trigger is also referred to as the 12 o’clock position. If the fenestration must avoid stent struts, then the fenestration is deemed to be at 12 o’clock, as is the position of the trigger relative to the stent graft. The fenestration can be created using scissors or a cautery device. The fenestration can be strengthened using the loop of a snare [14, 15]. To indicate the position of the fenestration during the DSA, either the original mark in the stent graft or an extra marker can be sutured to the fenestration [16]. After passing the aortic arch, the fenestration mark is aligned with the branch artery’s corresponding position. After deployment of the stent graft, a 0.018-inch guidewire is advanced through the fenestration. Deployment of stent in the branch artery.

Follow-up appointments are scheduled at 1, 6, and 12 months after surgery. An aortic CTA will be required for every follow-up.

### Criteria for discontinuing or modifying allocated interventions {11b}

The dropout criteria are as follows: (1) the participants request withdrawal; (2) the investigators consider it inappropriate to continue for safety concerns.

### Strategies to improve adherence to interventions {11c}

The participants in the experimental group will undergo surgical intervention promptly upon admission through research channels, which will help to improve adherence.

### Relevant concomitant care permitted or prohibited during the trial {11d}

Relevant concomitant care or intervention will be determined by physicians based on clinical practice guidelines at their discretion during the trial.

### Provisions for post-trial care {30}

Participants will be compensated for any injuries resulting from trial participation in accordance with the relevant laws of China.

### Outcomes {12}

In this trial, the outcomes will be developed according to the standard clinical endpoint of endovascular aortic repair [17]. The primary outcome will be the all-cause mortality (30 days). The secondary outcomes will include incidence of secondary intervention (30 days, 6 months, 1 year), incidence of endoleak (30 days, 6 months, 1 year), incidence of major adverse events (MAE) (i.e., immediate procedural success and complications) (30 days, 6 months, 1 year), immediate technical success rate, and all-cause mortality (6 months, 1 year). All-cause mortality is the annual number of deaths in a given group per the population in that group. Endoleak was assessed by DSA angiography during surgery and by CTA and DUS after surgery. We recommend re-intervening to achieve a proper seal in patients with type I or type III endoleaks following TEVAR according 2024 ESC Guidelines [18]. We recommend to perform 30-day imaging after TEVAR, by CTA and DUS, to assess the success of intervention. A secondary intervention will be performed if necessary, based on the follow-up results and the patient’s condition.

### Participant timeline {13}

See Fig. 2.

**Fig. 2:**
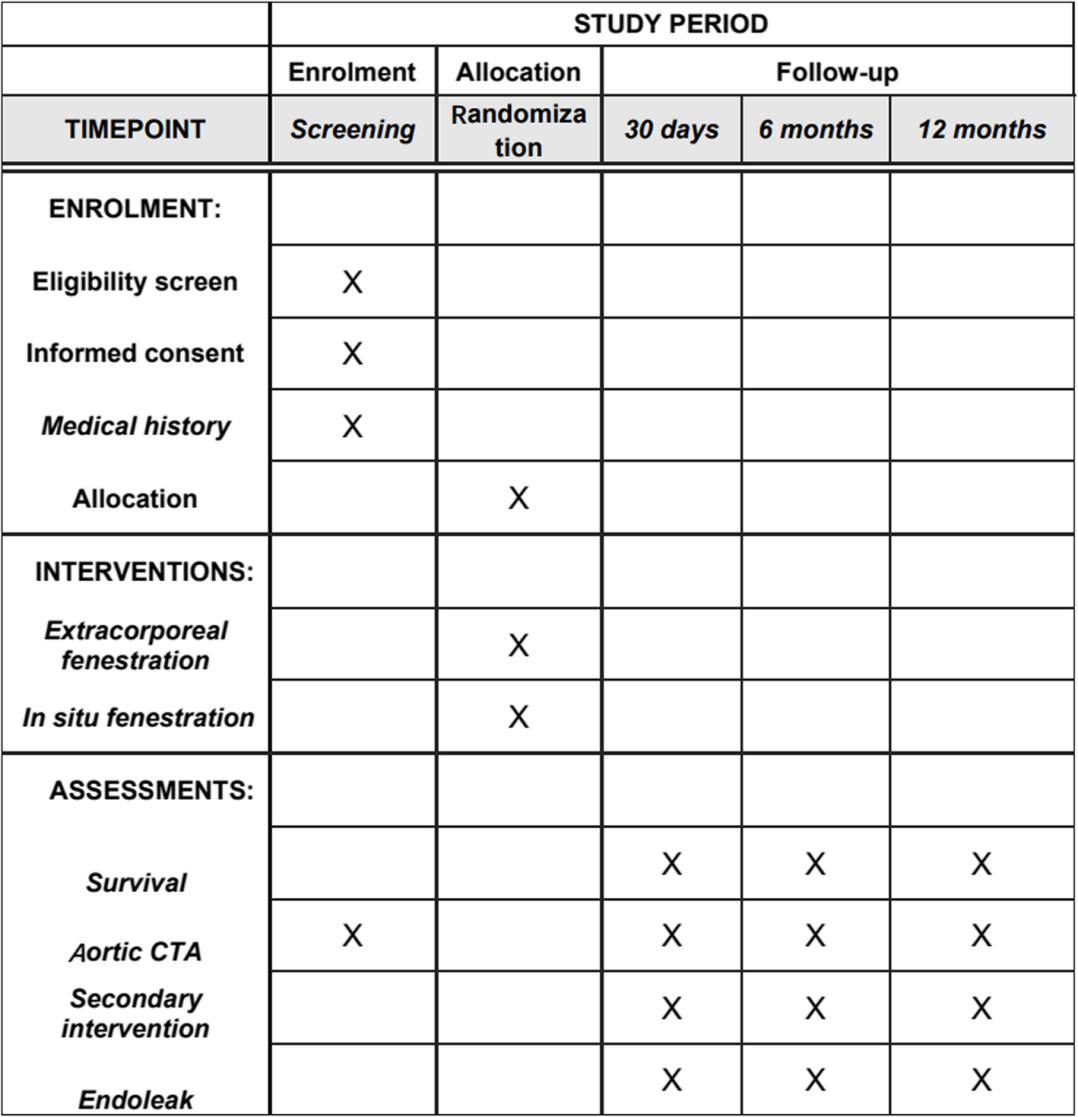
Standard Protocol Items: Recommendations for Interventional Trials (SPIRIT) enrollment schedule, interventions, and assessments

### Sample size {14}

The power of the trial is calculated as two co-primary endpoints, sharing a combined *α*-level of 0.05. According to the literature, cumulative 30-day all-cause mortality was similar in both groups: 3.8% [8]. An equal (1:1) equivalence design was used, with *α* = 0.05 (two-sided), *β* = 0.20 (two-sided), Δ = 10%, from which the sample size *n* was estimated. In accordance with the design of the trial and the primary efficacy outcome measures, the sample size was estimated using the following formula:

$$n=\frac{\pi_t\times\;\left(1-\pi_t\right)\;+\;\pi_c\;\times\;\left(1-\pi_c\right)}{\left(\mathit\triangle\mathit\;\mathit-\mathit\;\mathit{\left|{\pi_t-\pi_c}\right|}\right)^2}\times\left(\mu_{\mathit a\mathit/\mathit2}\mathit+\mu_{\mathit\beta}\right)^2$$

A minimum of 154 patients (77 per group) will need to be recruited. However, considering a potential dropout rate of 10%, combined with the low probability of adverse events in this trial, the total sample size was expanded to 170 patients.

### Recruitment {15}

Participants in this trial will be recruited from The Fourth Affiliated Hospital, Zhejiang University School of Medicine.

## Assignment of interventions: allocation

### Sequence generation {16a}

Patients will be randomized (using a random number table generated by the Excel RANDOM function) to group A or B. A fixed data analyst will generate the table of random numbers via computer.

### Concealment mechanism {16b}

The allocation sequence will be securely stored in sequentially numbered, opaque, sealed envelopes. Once the consent form is completed, the allocation will be revealed to the investigator.

### Implementation {16c}

The randomization scheme will be hidden from doctors and patients.

## Assignment of interventions: blinding

### Who will be blinded {17a}

Patients will be randomized (using a random number table generated by the Excel RANDOM function) to group A or B. A fixed data analyst will generate the table of random numbers via computer. The randomization scheme will be hidden from doctors and patients. Since the intervention in this trial could not blind the doctors, the data analyzers and patients were blinded.

### Procedure for unblinding if needed {17b}

Given the nature of our intervention, which involves extracorporeal fenestration and in situ fenestration, our study is designed as an unblinded trial.

## Data collection and management

### Plans for assessment and collection of outcomes {18a}

Baseline data will be collected after participants have signed informed consent. For the experimental group, intervention-related data will be obtained from the electronic medical records system within 30 days after patient discharge. Follow-up data will be obtained through outpatient visits or telephone interviews after the participants have completed each follow-up aortic CTA examination.

### Plans to promote participant retention and complete follow-up {18b}

A researcher will proactively remind participants of their scheduled CTA follow-up appointments via telephone.

### Data management {19}

Data will be collected in case report forms (CRF), including demographic characteristics, symptoms, signs, laboratory tests, medication, complications, and morphological outcomes. All the CRFs will be filled out independently by one researcher and checked by another researcher. All data collected during the study will be kept for 3 years after the completion of the study.

### Confidentiality {27}

Data access will be strictly limited to the designated study researchers. Any personal information collected during the trial will be treated with utmost confidentiality and handled in strict adherence to the pertinent legal provisions.

### Plans for collection, laboratory evaluation, and storage of biological specimens for genetic or molecular analysis in this trial/future use {33}

Not applicable; no biological specimens will be collected for this trial.

## Statistical methods

### Statistical methods for primary and secondary outcomes {20a}

Baseline characteristics and clinical outcomes will be expressed as numbers (percentages) for categorical variables and mean ± standard deviation for continuous variables. The chi-square test or Fisher’s exact test will be used to analyze categorical variables, and the independent *t*-test or the Wilcoxon signed-rank test will be adopted to analyze continuous variables. The Kaplan–Meier method will be performed to generate survival curves, and the log-rank analysis will be used to compare the differences between survival curves. Multivariate Cox regression adjusted by centers will be applied to calculate the hazard ratio risk. *P* < 0.05 will be considered to indicate statistical significance.

### Interim analyses {21b}

An interim analysis for efficacy of the primary endpoint will be done when 50% of planned sample size is assessed at 6 months. And it will give a recommendation whether the study should be continued or stopped.

### Methods for additional analyses (e.g., subgroup analyses) {20b}

Two prespecified subgroup analyses of age (18–49 vs. 50–80) and gender will be conducted for the primary outcome by adding an interaction term (e.g., age × group) into the Cox regression model. A sensitivity analysis will be conducted for the primary outcome based on the per-protocol set, which included patients who adhered to the treatment and follow-ups. Continuous data will be compared using the Student *t*-test or Mann–Whitney *U* test for parametric and non-parametric data, respectively. Categorical data will be compared using the chi-square test or Fisher’s exact test, as appropriate. Since the participants might crossover or be lost to follow-up, non-inferiority will be tested using two analysis sets. One set is the intention-to-treat (ITT) set, which includes all patients as randomized, regardless of whether they received the assigned treatment. The other is the per-protocol (PP) set. *P* < 0.05 will be considered statistically significant, and all data will be analyzed using SAS 9.2 software.

### Methods in analysis to handle protocol non-adherence and any statistical methods to handle missing data {20c}

The primary outcome will be analyzed according to the modified intent-to-treat principle, which will minimally exclude patients without receiving the assigned treatment or with missing primary outcome data. We propose declaring extracorporeal fenestration non-inferior to in situ fenestration in patients with aortic disease involving the left subclavian artery, only if shown to be non-inferior using both the “intention-to-treat” and “per-protocol” analysis sets.

### Plans to give access to the full protocol, participant-level data, and statistical code {31c}

Upon reasonable request, the corresponding author will provide access to the full protocol, participant-level data, and statistical code.

## Oversight and monitoring

### Composition of the coordinating center and trial steering committee {5d}

The coordinating center for this clinical trial will be The Fourth Affiliated Hospital, Zhejiang University School of Medicine. We will develop a trial steering committee (TSC) responsible for providing strategic guidance, monitoring the progress of the trial, and making critical decisions related to the trial protocol and overseeing the overall conduct of the study. The TSC will consist of two independent experts in vascular surgery and one independent statistician.

### Composition of the data monitoring committee, its role and reporting structure {21a}

We will also develop a data monitoring committee (DMC) to independently monitor trial data, ensure participant safety, and maintain data integrity and quality throughout the study. The DMC will comprise surgeons, radiologists, and statistician. The DMC meeting will be held half a year.

### Adverse event reporting and harms {22}

An adverse event (AE) is an untoward medical occurrence in a patient or clinical study subject, which may or may not be caused by the investigational device. All such events, whether expected or not, should be recorded. A severe adverse event (SAE) is an untoward and unexpected medical occurrence or effect that results in death or is life-threatening, explicitly referring to an event in which the subject was at risk of death at the time of the event. It does not refer to an event that hypothetically might have caused death if it were more severe, requires hospitalization or prolongation of existing inpatients’ hospitalization, results in persistent or significant disability or incapacity, or results from a congenital anomaly or congenital disability. All AEs should be reported as following procedures.

An SAE form should be completed and sent by fax or email to the chief investigator within 24 h. All SAEs should be reported to the Research Ethical Committee; the event was “related” (i.e., resulted from the administration of any of the research procedures) and “unexpected” (i.e., an event that is not listed in the protocol as an expected occurrence). Reports of related and unexpected SAEs should be submitted within 15 days of the chief investigator’s awareness of the event, using the NRES SAE form for non-IMP studies. Investigators should report any SAEs as required by Research Ethics Committee, sponsor, and/or Research and Development Office.

The investigator and sponsor will do their best to prevent possible injury caused by the design of this study. In case of any injury related to this study, the sponsor will bear the corresponding treatment costs and make compensation by national laws and regulations. If an adverse event caused by the investigational compression treatment causes harm, the clinical research institution and the investigator will provide timely and adequate treatment and management according to the routine clinical treatment.

### Frequency and plans for auditing trial conduct {23}

An independent auditing team will perform formal audit regularly to verify the integrity and reliability of the study data and ensure that the trial is conducted in compliance with ethical standards and protocol. The Project Management Group will meet monthly, or more frequently if required, to review progress, address issues related to data collection or the intervention, and implement necessary adjustments to study procedures. The Trial Steering Group will hold quarterly meetings to oversee the trial’s overall objectives, ensuring participant safety and data integrity. The Ethics Committee of The Fourth Affiliated Hospital, Zhejiang University School of Medicine will be informed of trial progress and significant developments, convening as necessary to review patient safety and protocol adherence. This structured oversight framework ensures rigorous monitoring and quality assurance throughout the trial process.

### Plans for communicating important protocol amendments to relevant parties (e.g., trial participants, ethical committees) {25}

Notifying sponsor first, then the PI will notify the center, and that a copy of the revised protocol will be sent to the PI to add to the Investigator Site File. Any deviations from the protocol will be fully documented using a breach report form, and the protocol will be updated in the clinical trial registry. All protocol revisions will be communicated to the ethics committee and update the protocol in the clinical trial registry as required.

## Dissemination plans {31a}

The research results of this trial will be reported in domestic and international academic conferences, as well as in peer-reviewed journals.

## Discussion

TEVAR for thoracic aortic pathologies involving the aortic arch provides a feasible and effective approach for such diseases and has been widely used both in the world. However, it still faces great challenges [10]. The Society for Vascular Surgery Practice Guidelines suggest routine preoperative revascularization in patients who need elective TEVAR where achievement of a proximal seal necessitates coverage of the left subclavian artery [8]. Extracorporeal fenestration and in situ fenestration techniques were initially conceived to overcome emergency or as a bail out, but in point of fact 60% or more of indications have turned out to be elective, echoing broad acceptance of the treatment and, one can suppose, its effectiveness [19, 20].

Several studies have demonstrated the safety and effectiveness of extracorporeal fenestration and in situ fenestration in patients with aortic disease involving the left subclavian artery [4–7]. However, extracorporeal fenestration or in situ fenestration techniques which is the best choice were unknown.

However, our study has several limitations. First, the surgeons cannot be blinded to the intervention. Second, the sample size is still relatively small, although we have calculated it. This was a single-center clinical trial, and multicenter clinical trials should be considered. Another limitation is that the postoperative follow-up time is short. Therefore, a worldwide large-scale clinical trial is still warranted in the future.

In summary, this study will compare the safety and validity of extracorporeal fenestration and in situ fenestration in patients with aortic disease involving the left subclavian artery. If in situ fenestration is non-inferior to extracorporeal fenestration in patients with aortic disease involving the left subclavian artery, this trial could contribute to future updates of clinical guidelines for these patients.

## Trial status

Study protocol version 1.0, dated January 7, 2024. Recruitment has not yet begun and is expected to begin on May 1, 2024.

## Data Availability

The data that support the findings of this study are available on request from the corresponding author, YB, upon reasonable request.

## Abbreviations

TEVAR: Thoracic EndoVascular Aortic Repair
LSA: Left subclavian artery
LCCA: Left common carotid artery
LBA: Left brachial artery
CTA: Computed tomography angiography
CRF: Case report forms
DMC: Data monitoring committee
AE: Adverse event
SAE: Severe adverse event

## References

[1] Spath P, Campana F, Tsilimparis N, et al. Outcomes of fenestrated and branched endografts for partial and total endovascular repair of the aortic arch - a systematic review and meta-analysis. Eur J Vasc Endovasc Surg. Published online August 2, 2023. 10.1016/j.ejvs.2023.07.048. 10.1016/j.ejvs.2023.07.048 37536517

[2] Al-Hakim R, Schenning R. Advanced techniques in thoracic endovascular aortic repair: chimneys/periscopes, fenestrated endografts, and branched devices. Tech Vasc Interv Radiol. 2018;21(3):146–55. 10.1053/j.tvir.2018.06.004. 30497549 10.1053/j.tvir.2018.06.004

[3] Anwar MA, Hamady M. Various endoluminal approaches available for treating pathologies of the aortic arch. Cardiovasc Intervent Radiol. 2020;43(12):1756–69. 10.1007/s00270-020-02561-y. 32588136 10.1007/s00270-020-02561-y PMC7649180

[4] Pinheiro Ribeiro Alves A, da Costa Cettolin Q, Dos Santos Domingues G, Dos Santos HÁG, Sampaio RC, Aquino MA. Results of the in situ fenestration technique for preservation of the left subclavian artery in the endovascular repair of acute aortic syndromes in a reference center. Vascular. 2023:17085381231155959. 10.1177/17085381231155959. Epub ahead of print. PMID: 36750245. 10.1177/17085381231155959 36750245

[5] Yu Z, Hu S, Wang D, Yang T, Lang D. Early and midterm outcomes of in situ fenestration via adjustable puncture needle for Ankura aortic stent graft: a single-center experience. Vascular. 2023:17085381231192376. 10.1177/17085381231192376. Epub ahead of print. PMID: 37496151. 10.1177/17085381231192376 37496151

[6] Redlinger RE Jr, Ahanchi SS, Panneton JM. In situ laser fenestration during emergent thoracic endovascular aortic repair is an effective method for left subclavian artery revascularization. J Vasc Surg. 2013;58(5):1171–7. 10.1016/j.jvs.2013.04.045. (Epub 2013 Jun 5 PMID: 23746832). 23746832 10.1016/j.jvs.2013.04.045

[7] Kuo HS, Huang JH, Chen JS. Handmade stent graft fenestration to preserve left subclavian artery in thoracic endovascular aortic repair†. Eur J Cardiothorac Surg. 2019;56(3):587–94. 10.1093/ejcts/ezz049. (PMID: 30809647). 30809647 10.1093/ejcts/ezz049

[8] Boufi M, Alexandru G, Tarzi M, Zlitni M, Taghi H, Loundou AD. Systematic review and meta-analysis of ex-situ and in-situ fenestrated stent-grafts for endovascular repair of aortic arch pathologies. J Endovasc Ther. Published online March 10, 2023. 10.1177/15266028231157639. 10.1177/15266028231157639 36896884

[9] Canonge J, Heim F, Chakfé N, Coscas R, Cochennec F, Jayet J. Mechanical performance assessment of physician modified aortic stent graft. Eur J Vasc Endovasc Surg. 2023;65(3):435–43. 10.1016/j.ejvs.2022.11.004. 36343747 10.1016/j.ejvs.2022.11.004

[10] Qiu C, Li Z, Dai X, et al. Technical details of thoracic endovascular aortic repair with fenestrations for thoracic aortic pathologies involving the aortic arch: a Chinese expert consensus. Front Cardiovasc Med. 2022;9:1056229. Published 2022 Dec 20. 10.3389/fcvm.2022.1056229. 10.3389/fcvm.2022.1056229 PMC9807668 36606283

[11] Li Y, He C, Chen X, Yao J, Zhang T, Zhang H. Endovascular in situ fenestration technique of aortic arch pathology: a systematic review and meta-analysis. Ann Vasc Surg. 2021;76:472–80. 10.1016/j.avsg.2020.12.021. 33508460 10.1016/j.avsg.2020.12.021

[12] Xiang Y, Qiu C, He Y, et al. A single center experience of in situ needle fenestration of supra-aortic branches during thoracic endovascular aortic repair. Ann Vasc Surg. 2019;61:107–15. 10.1016/j.avsg.2019.03.01. 31200061 10.1016/j.avsg.2019.03.016

[13] Shang T, Tian L, Li DL, Wu ZH, Zhang HK. Favourable outcomes of endovascular total aortic arch repair via needle based in situ fenestration at a mean follow-up of 5.4 months. Eur J Vasc Endovasc Surg. 2018;55(3):369–376. 10.1016/j.ejvs.2017.11.022. 10.1016/j.ejvs.2017.11.022 29306627

[14] Zhang L, Wu MT, Zhu GL, et al. Off-the-shelf devices for treatment of thoracic aortic diseases: midterm follow-up of TEVAR with chimneys or physician-made fenestrations. J Endovasc Ther. 2020;27(1):132–42. 10.1177/1526602819890107. 31789078 10.1177/1526602819890107

[15] Zhu J, Dai X, Noiniyom P, et al. Fenestrated thoracic endovascular aortic repair using physician-modified stent grafts (PMSGs) in zone 0 and zone 1 for aortic arch diseases. Cardiovasc Intervent Radiol. 2019;42(1):19–27. 10.1007/s00270-018-2079-9. 30327926 10.1007/s00270-018-2079-9

[16] Miura S, Kurimoto Y, Maruyama R, et al. Thoracic endovascular aortic repair on zone 2 landing for type B aortic dissection. Ann Vasc Surg. 2019;60:120–7. 10.1016/j.avsg.2019.02.017. 31075454 10.1016/j.avsg.2019.02.017

[17] Diehm N, Vermassen F, van Sambeek MR; DEFINE Investigators. Standardized definitions and clinical endpoints in trials investigating endovascular repair of aortic dissections. Eur J Vasc Endovasc Surg. 2013;46(6):645–650. 10.1016/j.ejvs.2013.08.017. 10.1016/j.ejvs.2013.08.017 24076081

[18] Mazzolai L, Teixido-Tura G, Lanzi S, et al. 2024 ESC guidelines for the management of peripheral arterial and aortic diseases. Eur Heart J. 2024;45(36):3538–700. 39210722 10.1093/eurheartj/ehae179

[19] Matsumura JS, Lee WA, Mitchell RS, et al. The Society for Vascular Surgery practice guidelines: management of the left subclavian artery with thoracic endovascular aortic repair. J Vasc Surg. 2009;50(5):1155–8. 10.1016/j.jvs.2009.08.090. 19878791 10.1016/j.jvs.2009.08.090

[20] Murphy EH, Dimaio JM, Dean W, Jessen ME, Arko FR. Endovascular repair of acute traumatic thoracic aortic transection with laser-assisted in-situ fenestration of a stent-graft covering the left subclavian artery. J Endovasc Ther. 2009;16(4):457–63. 10.1583/09-2746.1. 19702349 10.1583/09-2746.1

